# A Case of the Nutcracker Syndrome Developed after Delivery

**DOI:** 10.1155/2014/503017

**Published:** 2014-07-22

**Authors:** Koji Tsumura, Kanae Yoshida, Sachi Yamamoto, Sayuri Takahashi, Katsuyuki Iida, Yutaka Enomoto

**Affiliations:** Department of Urology, Mitsui Memorial Hospital, Kanda Izumi-cho 1, Chiyoda-ku, Tokyo 101-8643, Japan

## Abstract

We report a case of nutcracker syndrome that developed after delivery. A 32-year-old woman visited our clinic complaining of gross hematuria 4 months after delivery. Urethrocystoscopic examination failed to show hematuria coming from the ureteral orifice; however, enhanced computed tomography revealed the compression of the left renal vein between the aorta and superior mesenteric artery. Therefore, we diagnosed her with nutcracker syndrome and conservatively observed her. The macrohematuria disappeared by itself after 1 month. This is the first report to describe a case of nutcracker syndrome that developed after delivery.

## 1. Introduction

Nutcracker syndrome is caused by the compression of the left renal vein (LRV) between the superior mesenteric artery (SMA) and aorta [1]. The classical symptoms include left flank pain with gross or microscopic hematuria. Some patients with severe symptoms have been treated using surgical procedures. However, patients with mild symptoms can be conservatively managed until collateral veins develop and the LRV hypertension is resolved. Some reports have suggested that pregnancy can worsen symptoms, but no cases of nutcracker syndrome that developed after pregnancy have been reported. To the best of our knowledge, this is the first report of nutcracker syndrome that developed after pregnancy.

## 2. Case Presentation

**Figure 1:**
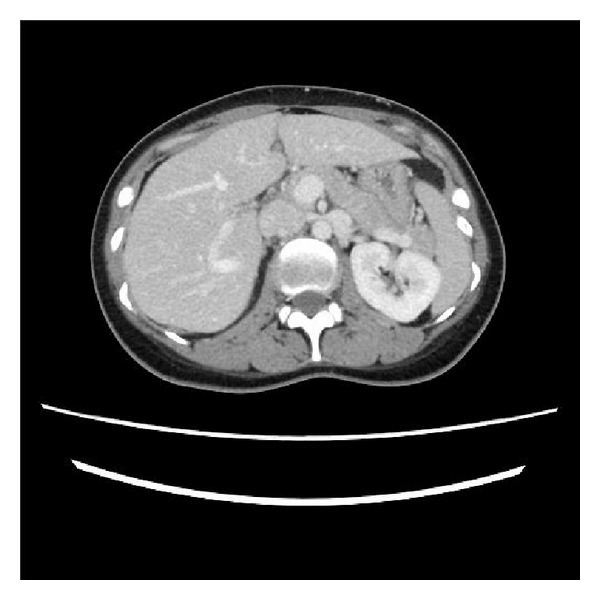
Enhanced computed tomography of transverse section. Left renal vein becomes compressed between the superior mesenteric artery and the aorta.

A 32-year-old parous woman visited our clinic complaining of gross hematuria. Before 4 months, her second pregnancy normally went to term and a healthy infant was delivered in the 39th week of gestation via vaginal delivery. Urinalysis revealed a urinary-specific gravity of 1.037, 100 mg/dL proteins, and numerous red blood cells per high power field. The result of urine cytology was class 1. Laboratory tests revealed no abnormal findings. Urethrocystoscopic examination failed to show hematuria coming from the ureteral orifice. Ultrasonography revealed no abnormal findings in the ureters and kidneys. Enhanced computed tomography (CT) demonstrated the compression of the LRV between the aorta and SMA with marked dilation of LRV and the venous collaterals (Figure 1). Angiography to measure the pressure gradient was not performed. The diagnosis of nutcracker syndrome was made on the basis of these findings. Therefore, the patient was conservatively observed. The macrohematuria disappeared by itself after 1 month.

## 3. Discussion

Nutcracker syndrome occurs because of the compression of LRV between SMA (anteriorly) and the abdominal aorta (posteriorly). It has been postulated that the increased venous pressure ruptures the thin-walled septum between the small veins and collecting system in the renal fornix, resulting in hematuria [2].

**Figure 2:**
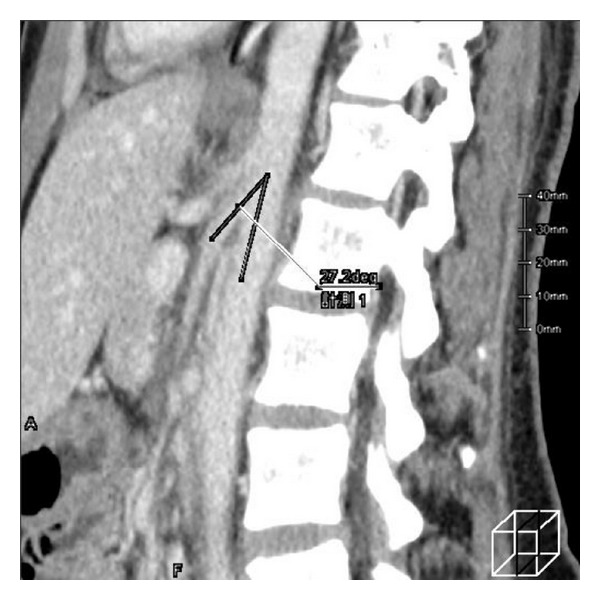
Enhanced computed tomography of sagittal section. The angle between the aorta and the SMA was 27.2 degrees.

No clinical diagnostic criteria exist, and most patients are diagnosed only after the exclusion of other causes of flank pain or hematuria. Ishidoya et al. suggested that the following conditions were required for diagnosis: (1) there is no urological disease associated with hematuria and flank pain, (2) the distance between SMA and aorta is <5 mm, (3) the maximum blood flow velocity of LRV is <15 mL/sec, and (4) the pressure gradient between the distal renal vein and inferior vena cava is >4 cm H_2_O in the supine position [3]. Shokeir et al. performed a sagittal CT study and concluded that the angle between the aorta and SMA was more acute (42°–51°) in three patients with nutcracker syndrome than the 90° angle observed in normal subjects [4]. In our patient, contrast enhanced CT explained the severe compression of LRV. The distance between SMA and the aorta was 4 mm, and the angle between the aorta and SMA was 27° (Figure 2). Therefore, she was diagnosed with probable nutcracker syndrome that resulted in hematuria. Because of the mild symptoms, we determined that more invasive procedures such as venography and measuring the renocaval pullback pressure gradient were not necessary.

Some cases of nutcracker syndrome during pregnancy have been reported [5, 6]; however, few reports have suggested a relationship between symptoms and pregnancy. One report attributed the gross hematuria to an increase in renal plasma flow during pregnancy [5]. In this case, the gross hematuria disappeared after delivery by Cesarean section, and the diagnosis of nutcracker syndrome was made after venography on postpartum day 16 and CT on postpartum day 25. Therefore, this suggested that the imaging abnormalities that occur during nutcracker syndrome remain for approximately 1 month after delivery.

We made the following assumption about etiology of nutcracker syndrome of our patient: the involution of the uterus resulted in caudal traction of SMA, which compressed LRV leading to venous hypertension of LRV even though the renal plasma flow was restored to prepregnancy values. However, this assumption has limitations because the involution of the uterus is complete within 6 weeks of delivery, but our patient developed symptoms 4 months after delivery. Therefore, it is possible that she developed nutcracker syndrome by chance.

Several treatment options exist for nutcracker syndrome including observation; however, treatment remains controversial. Several surgical approaches have been previously described, including Gore-Tex graft vein interposition, transposition of the left renal vein [7], nephropexy, stenting, renocaval reimplantation, and renal autotransplantation [8, 9]. However, these surgical approaches are invasive and are not established as the gold standard. Although observation is noninvasive, some reports suggested that it took over 3 years to develop collateral veins; therefore, it is unclear how long the symptoms last [10]. We conservatively observed our patient because her symptoms were mild. Careful surveillance will be needed as other diseases causing macrohematuria are not completely ruled out.
